# Genome-wide diversity and runs of homozygosity in the “Braque Français, type Pyrénées” dog breed

**DOI:** 10.1186/s13104-017-3112-9

**Published:** 2018-01-09

**Authors:** Salvatore Mastrangelo, Filippo Biscarini, Barbara Auzino, Marco Ragatzu, Andrea Spaterna, Roberta Ciampolini

**Affiliations:** 1 Dipartimento di Scienze Agrarie e Forestali, Università di Palermo, Palermo, Italy; 2 CNR-IBBA, Via Bassini 15, 20133 Milano, Italy; 3 Club Italiano Braque Français Type Pyrénées, Capalbio, GR Italy; 4 Scuola di Scienze Mediche Veterinarie, University of Camerino, Matelica, MC Italy; 5 Centro Interuniversitario di Ricerca e di Consulenza sulla Genetica e la Clinica del cane, Matelica, MC Italy; 6 Dipartimento di Scienze Veterinarie, Università di Pisa, V.le delle Piagge 2, 56124 Pisa, Italy

**Keywords:** Dog, Braque Français, type Pyrénées, SNP, Genetic diversity, Molecular markers, Inbreeding, Runs of homozygosity, Heterozygosity

## Abstract

**Objective:**

Braque Français, type Pyrénées is a French hunting-dog breed whose origin is traced back to old pointing gun-dogs used to assist hunters in finding and retrieving game. This breed is popular in France, but seldom seen elsewhere. Despite the ancient background, the literature on its genetic characterization is surprisingly scarce. A recent study looked into the demography and inbreeding using pedigree records, but there is yet no report on the use of molecular markers in this breed. The aim of this work was to genotype a population of Braque Français, type Pyrénées dogs with the high-density SNP array to study the genomic diversity of the breed.

**Results:**

The average observed ($H_O$) and expected ($H_E$) heterozygosity were 0.371 ($\pm \,0.142$) and 0.359 ($\pm \,0.124$). Effective population size ($N_e$) was 27.5635 runs of homozygosity (ROH) were identified with average length of 2.16 MB. A ROH shared by $75\%$ of the dogs was detected at the beginning of chromosome 22. Inbreeding coefficients from marker genotypes were in the range $F_{IS}=[-\,0.127,0.172]$. Inbreeding estimated from ROH ($F_{ROH}$) had mean $0.112\,(\pm \,0.023$), with range [0.0526, 0.225]. These results show that the Braque Français, type Pyrénées breed is a relatively inbred population, but with still sufficient genetic variability for conservation and genetic improvement.

**Electronic supplementary material:**

The online version of this article (10.1186/s13104-017-3112-9) contains supplementary material, which is available to authorized users.

## Introduction

Genetic variability and structure in domestic animals largely depend on breeders’ decisions and practices. In selection, breeding within a closed population is common practice, since it allows to fix the desired characteristics and traits of the best representatives of the breed. However, this mating practice can lead to high rates of inbreeding and associated risks (higher frequency of recessive disorders, inbreeding depression), which are a serious threat especially to small populations and to populations originating from a small number of ancestors [1, 2]. Furthermore, intense directional selection for specialized animal types may result in a reduced genetic basis available to the populations, which leads to a dramatic loss of genetic variability, especially in dogs where mating between close relatives is frequently used [3, 4]. Concerns about the potential effects of inbreeding and reduced diversity on health, functionality and welfare of animals within dog breeds, have led to a call for improved genetic management practices. Hence, managing genetic diversity has become a major focus for dog breeders, herd books and authorities [5]. Studies have shown that there is a loss of the total amount of genetic diversity in modern dog populations [6–8]. In particular, purebred dogs have been intensely selected by resorting, in some lines, to close-breeding where popular dominant sires were repeatedly used for mating, resulting in a reduction of genetic diversity. Traditionally, genealogical data has been used to assess genetic diversity in dogs [3–5, 9, 10]. However, the use of genealogical data is limited by the incomplete or inaccurate available pedigree records. Genomics offer novel applications that have great potential to increase our understanding of the genome of domestic animals and to improve the efficiency of conservation and selection programs [11–13]. STR (short-tandem repeats) molecular markers have been used initially to estimate genetic diversity in the absence of pedigree records [9, 10, 14, 15]. More recently, the availability of high density single nucleotide polymorphism (SNP) arrays, has increased the accuracy, the throughput and the cost-effectiveness of genomic analyses for conservation genetics [16, 17]. Indeed, the large numbers of SNPs throughout the genome makes these markers particularly suitable for the detection of genomic regions where a reduction in heterozygosity occurred and offers new opportunities to improve the accuracy of genetic diversity estimates.

Braques Françaises are hunting dogs, originating from a very old type of gun-dog used for pointing the location of game birds to hunters. There are two breeds of Braque Français, both from the south of France: the Braque Français, type Gascogne (larger size) and the Braque Français, type Pyrénées (smaller size). They are popular hunting dogs in France, but are seldom seen elsewhere. The original Braque Français type of pointing dog has existed since the eighteenth century. The first breed club was formed in 1850, and the standards for both breeds were written in 1880. The demography and inbreeding levels of the Braque Français, type Pyrénées breed were estimated using pedigree records [18]. There is yet no report of the genetic characterization of this breed using molecular markers. The aim of this work was to study the genomic diversity of Braque Français, type Pyrénées dogs using data high-density SNP data. Available data, methods and results are hereby presented.

## Main text

### DNA sampling, genotyping and quality control

Blood samples were collected from 48 Braque Français, type Pyrénées unrelated individual dogs (27 females, 21 males), to capture a representative sample of the within-breed genetic diversity. Genomic DNA (gDNA) was extracted from blood samples through a standard ethanol fractionation with concentrated sodium chloride (6 M NaCl) and sodium dodecyl sulphate (10% SDS). The concentration of DNA was adjusted to 50 ng/$\upmu$L per sample. All dogs were genotyped with the Illumina CanineHD BeadChip, containing 173,662 SNPs. Genotyping was carried out in the laboratories of “Dipartimento di Scienze Agrarie, Alimentari e Forestali”, University of Palermo (Italy). SNP data were filtered to exclude unmapped loci not assigned to any chromosomes and loci on the sex chromosomes: only SNPs located on the 38 autosomes were considered. Additionally, SNPs with call-rate $< 95\%$ and minor allele frequency (MAF) $<5\%$ were removed from the dataset, as well as animals with over $10\%$ missing genotypes.

### Genetic diversity parameters, effective population size and runs of homozygosity (ROH)

Basic genetic diversity indices were estimated, including: (i) observed heterozygosity ($H_O=\frac{1}{L}\sum _{l=1}^L \left( \frac{n_{AB}}{N}\right)$); (ii) expected heterozygosity ($H_E=\frac{1}{L}\sum _{l=1}^L \left( 1-p_l^2 - q_l^2 \right)$); (iii) the inbreeding coefficient based on the difference between observed and expected homozygous genotypes ($F_{IS}=\frac{1}{L}\sum _{l=1}^L \left( 1-\frac{H_{Ol}}{H_{El}} \right)$, [19]); and (iv) minor allele frequency ($MAF_l= \frac{n_{B_l}}{2N_l}$). Here: *L* is the n. of SNP loci; $n_{AB}$ is the number of heterozygous genotypes at each locus *l*; *N* is the number of individuals (sample size); *p* and *q* are the frequencies of, respectively, the *A* (major) and *B* (minor) alleles.

The contemporary effective population size ($N_e$) was estimated based on linkage disequilibrium (LD; [20]). To reduce the impact of SNP ascertainment bias from linkage between loci, unlinked SNPs were selected based on variance inflation factor (VIF, a measure of multicollinearity in multiple regression) below 2 in 50-SNP sliding window with 5-SNP step. A VIF threshold of 2 is recommended for small sample sizes to avoid removing too many SNPs (http://zzz.bwh.harvard.edu/plink/summary.shtml#prune).

Runs of homozygosity (ROH: segments of continuous homozygous genome, [21]) were detected in each individual dog using the sliding-window based method described in [22, 23]. A 50-SNP long sliding window was used to scan the genome; the proportion of overlapping homozygous windows to call a ROH was 0.05; maximum two missing and one heterozygous SNP were allowed in a run; the minimum number of SNPs to call a ROH (*s*) was calculated as proposed by Lencz et al. [24] to minimize the number of false positive ROH:

1 $$\begin{aligned} s=\frac{\ln \frac{\alpha }{N \cdot L}}{\ln (1-\overline{H_O})} \end{aligned}$$

where $\alpha$ is the tolerated proportion of false positive ROH (0.05 in the present study); *N* and *L* are the sample size and number of SNP loci; $\overline{H_O}$ is the average observed heterozygosity across individuals and SNPs. The minimum length of a ROH was set to ${1}\mathrm{\,MB}$ to exclude short ROH arising as a consequence of linkage disequilibrium (LD) [5]. Additionally, a minimum density of one SNP every 50 kB and a maximum 100 kB gap between consecutive SNPs were required to define a ROH.

The inbreeding coefficient based on ROH ($F_{ROH}$) for each animal was estimated as the ratio between the sum of the length of all ROH ($L_{ROH}$) and the total length of the autosomal genome covered by SNPs on the array ($L_{AUTO} = {2268.83}\mathrm{\,MB}$).

2 $$\begin{aligned} F_{ROH}=\frac{\sum L_{ROH}}{L_{AUTO}} \end{aligned}$$

### Software

Quality control filtering of genotypic data, ROH detection and the estimation of genetic parameters were all performed using the *PLINK* software package [22]. $N_e$ was estimated using NeEstimator v.2 [25], with unlinked SNP selected using *PLINK*. The *R* programming environment for statistical computing v.3.2.3 [26] was used for data manipulation, summary statistics, preparation of tables and figures. The specific *PLINK* command lines are detailed in Additional file 1.

### Results

After filtering for quality (call-rate, genome assembly), the final number of SNPs retained for the analysis was 94,065. All 48 dogs had high quality genotyping and were included in the analysis. The average observed ($H_O$) and expected ($H_E$) heterozygosities were 0.371 ($\pm \,0.142$) and 0.359 ($\pm \,0.124$), respectively; the average MAF was 0.269 ($\pm \,0.132$). The $H_O$ and $H_E$ values reported here are comparable with previous studies that examined the genetic diversity in dogs: Pollinger et al. [27] and Pilot et al. [12] estimated average $H_O$ between 0.2 and 0.3 in modern dog breeds [12, 27]. Pertoldi et al. [6] reported $H_E$ estimates in five Danish dog breeds similar to the expected heterozygosity found here in Braque Français, type Pyrénées dogs. The reported MAF values also were consistent with the range found in literature [11, 28]. Recently, Stronen et al. [29] reported lower values of genetic diversity in the endangered Norwegian Lundehund (e.g. $H_O=0.038$) compared to three reference breeds. In the same breed, a very low MAF (0.033) has been estimated [7]. In comparison, the genetic diversity levels reported here for Braque Français, type Pyrénées dogs seem to indicate that this is not an endangered breed. The 5560 SNPs on the sex chromosomes were not used in this study, because of potentially ambiguous heterozygous SNP calls in males. As an indication, we report basic SNP descriptive statistics in males and females. Monomorphic SNPs on the sex chromosomes were 41.2 and $37.3\%$ in males and females respectively, with MAF 0.145 and 0.151, and missing-rate 0.0301 and 0.0293.

The estimated contemporary effective population size ($N_e$) in Braque Français, type Pyrénées dogs was 27; this value indicates a potential risk of inbreeding and reduction in genetic diversity. In a recent a study on Bullmastiff dogs, a similar $N_e$ value (29.1) was estimated using the same method [5]. The authors reported that a small $N_e$ is a reflection of population size, unequal use of breeding animals and unequal founder contributions.

**Fig. 1:**
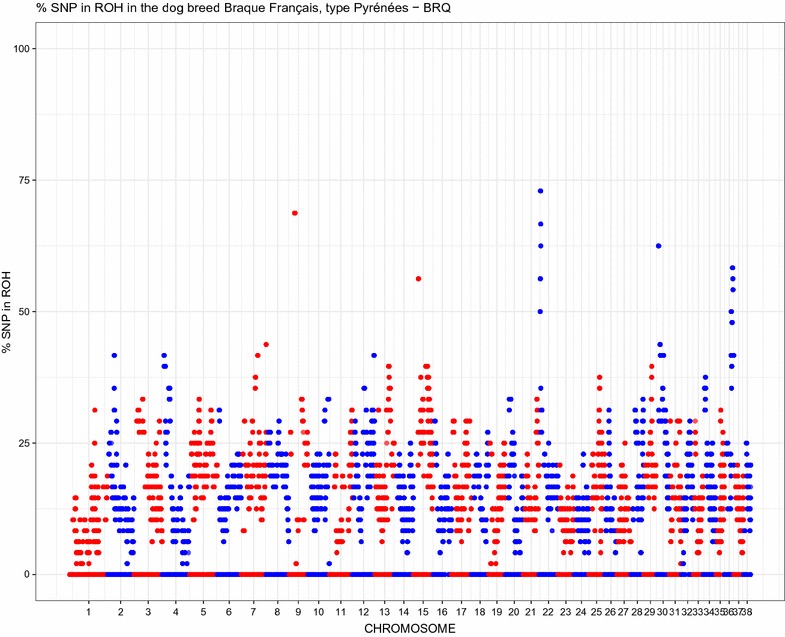
Manhattan plot of the proportion of times each SNP falls within a ROH in the analysed dog population

**Fig. 2:**
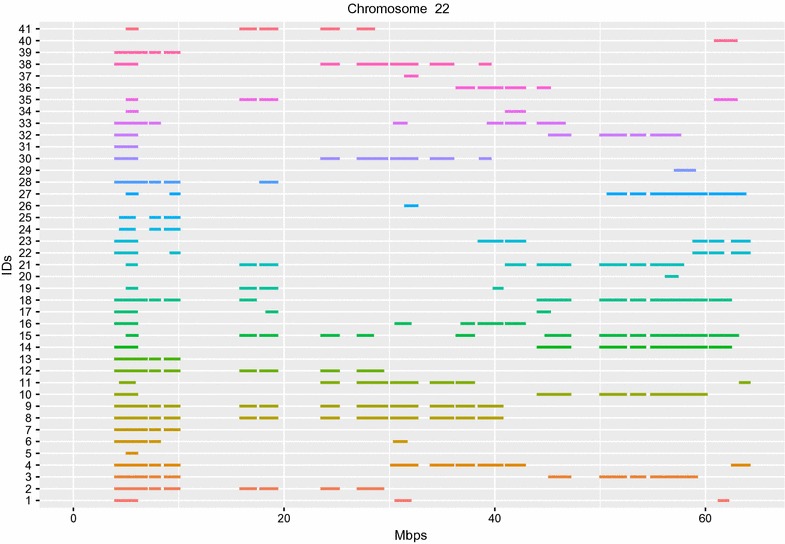
Runs of homozygosity (ROH) detected in each Braque Français, type Pyrénées dog on chromosome 22

A total of 5635 ROH were identified with an average of 117.38 ROH per dog (range between 52 and 230 ROH per animal). Table 1 reports the number of ROH and their average length per length class (0–2, 2–4, 4–8, 8–16 MB). The average number of ROH in Mediterranean dog breeds ranged from 12 (Mastino Abruzzese) to 114 (Saint Bernard) [30]: this positions the Braque Français, type Pyrénées in the higher part of the range. The longest ROH (12.5 MB) was found on chromosome 16. Figure 1 shows the proportion of times (dogs) each SNP falls inside a ROH, plotted against the SNP position along the dog genome. Especially on chromosome 22 there are a number of SNP loci which appear to fall relatively often within a ROH in the Braque Français, type Pyrénées dog breed. Indeed, at the beginning of chromosome 22 (5–10 MB) there is a ROH shard by most of the dogs in the analysed sample (Fig. 2).

**Table 1:** ROH counts and average length per class (MBps)

	Class (MBps)	Group	nRuns	Avg length (MBps)
1	1–2	BRQ	3390	1.43
2	2–4	BRQ	1752	2.67
3	4–8	BRQ	463	5.16
4	8–16	BRQ	29	9.22

*BRQ* Braque Français, type Pyrénées

Inbreeding coefficients estimated from SNP marker loci as $F_{IS}$ were in the range $[-\,0.127,0.172]$: some negative values were obtained, which corresponded to dogs with lower than average homozygosity. Inbreeding estimated from runs of homozygosity ($F_{ROH}$) had a mean of $0.112\,(\pm \,0.023$), with a range between 0.0526 to 0.225. In German shepherd dogs, similar $F_{ROH}$ values have been reported [8]. $F_{ROH}$ from 0.061 (Jack Russell Terrier) to 0.151 (Bulldog) was estimated in a panel of canine breeds [5]. The Pearson linear correlation between the two measures of molecular inbreeding ($F_{IS}$ and $F_{ROH}$ was 0.91. Previously, from pedigree records an average genealogical inbreeding coefficient (*F*, [31]) of $4.3\%$ was estimated [18].

## Limitations

The results presented in this note constitute the first report on the genomic characterization of the Braque Français, type Pyrénées dog breed using molecular markers. Previously, the demographics and diversity of this breed had been analysed resorting to pedigree data exclusively. The use of molecular data represents an important step towards a more accurate description of the genome of the Braque Français, type Pyrénées dogs. This improved genomic information will prove to be very useful for the management of the breed, both in the perspective of conservation and in that of breeding and selection. However, this is but a preliminary study, that aimed at providing essential initial information to be used subsequently for a larger and more comprehensive studies. Genomic data on a panel of relevant dog breeds are currently being acquired, to be used for a large-scale genome-wide characterization of dog breeds and the genetic distances and phylogenetic relationships between them. Additionally, phenotypes on the hunting ability and morphology of Braque Français, type Pyrénées dogs are being collected: together with the already available genotypic data, these phenotypes will be used in genome-wide association studies (GWAS) and similar approaches (e.g. see [32]) to detect SNP loci and regions of the genome that play a role in relevant phenotypes for the breed. As an illustration, the strong ROH signal detected on chromosome 22 may be associated to hunting ability or other phenotypic characteristics of the Braque Français, type Pyrénées dog breed. Furthermore, genomic data can be used to improve the accuracy of pedigree records, thereby allowing for more meaningful comparisons between genealogic and molecular inbreeding. All together, the results presented here provide an interesting example of the use of molecular markers to understand the genetic background and history of small canine breeds like the Braque Français, type Pyrénées.

## Additional file

**Additional file 1.** Plink command lines. File with the Plink command lines used to: (i) edite the SNP data; (ii) select unlinked SNP loci for the estimation of $N_e$; (iii) detect runs of homozygosity (ROH).

## Abbreviations

SNP: single nucleotide polymorphisms
ROH: runs of homozygosity
