# Supplementary material for: Genome-wide diversity and runs of homozygosity in the “Braque Français, type Pyrénées” dog breed
Source: BMC Res Notes. 2018 Jan 9;11:13. doi: 10.1186/s13104-017-3112-9 (PMC5761150; doi:10.1186/s13104-017-3112-9)
Supplement: Supplementary file 1 — Additional file 1. Plink command lines. File with the Plink command lines used to: (i) edite the SNP data; (ii) select unlinked SNP loci for the estimation of $N_e$Ne; (iii) detect runs of homozygosity (ROH). [file 13104_2017_3112_MOESM1_ESM.pdf]

RESEARCH

Genome-wide diversity and runs of homozygosity in the “Braque Francais, type Pyrénées” dog breed

Salvatore Mastrangelo[1†], Filippo Biscarini[2*†], Barbara Auzino[5], Marco Ragatzu[3], Andrea Spaterna[4,5] and Roberta Ciampolini[5,6]

*Correspondence: filippo.biscarini@ibba.cnr.it
[2]Dipartimento di Scienze Agrarie e Forestali - Università di Palermo, , Palermo, Italy
Full list of author information is available at the end of the article
†Equal contributorˆ
salvatore.mastrangelo@unipa.it
barbara.auzino@yahoo.it
marcoragatzu@gmail.com
andrea.spaterna@unicam.it
roberta.ciampolini@unipi.it

Additional File 1 - Plink command lines [1]

PLINK command lines for SNP data editing

```
--dog
--chr 1-38
--geno 0.05
--mind 0.10
--maf 0.05
```

PLINK command lines to select unlinked SNP loci

```
--dog
--indep 50 5 2
```

PLINK command lines for ROH

```
--dog
--homozyg-window-het 1
--homozyg-snp 45
--homozyg-window-snp 50
--homozyg-window-missing 2
--homozyg-window-threshold 0.05
--homozyg-kb 1000
--homozyg-density 50
--homozyg-gap 100
```

Author details
[1]CNR-IBBA, Via Bassini 15, 20133 Milano, Italy. [2]Dipartimento di Scienze Agrarie e Forestali - Università di Palermo, , Palermo, Italy. [3]Club Italiano Braque Français type Pyrénées, Capalbio (GR), Italy. [4]Scuola di Scienze Mediche Veterinarie, University of Camerino, Matelica (MC), Italy. [5]Centro Interuniversitario di Ricerca e di Consulenza sulla Genetica e la Clinica del cane, Matelica (MC), Italy. [6]Dipartimento di Scienze Veterinarie - Università di Pisa, V.le delle Piagge 2, 56124 Pisa, Italy.

References

1. Purcell, S., Neale, B., Todd-Brown, K., Thomas, L., Ferreira, M.A., Bender, D., Maller, J., Sklar, P., De Bakker, P.I., Daly, M.J., *et al.*: Plink: a tool set for whole-genome association and population-based linkage analyses. The American Journal of Human Genetics **81**(3), 559–575 (2007)
